# Strontium isotope analysis on cremated human remains from Stonehenge support links with west Wales

**DOI:** 10.1038/s41598-018-28969-8

**Published:** 2018-08-02

**Authors:** Christophe Snoeck, John Pouncett, Philippe Claeys, Steven Goderis, Nadine Mattielli, Mike Parker Pearson, Christie Willis, Antoine Zazzo, Julia A. Lee-Thorp, Rick J. Schulting

**Affiliations:** 1 School of Archaeology, University of Oxford, 1-2 South Parks Road, Oxford, OX1 3TG UK; 2 Analytical, Environmental & Geo-Chemistry, Dept. of Chemistry, Vrije Universiteit Brussel, AMGC-WE-VUB, Pleinlaan 2, 1050 Brussels, Belgium; 3 G-Time Laboratory, Université Libre de Bruxelles, CP 160/02, 50, Avenue F.D. Roosevelt, B-1050 Brussels, Belgium; 4 Institute of Archaeology, University College London, 31-34 Gordon Square, WC1H 0PY London, UK; 5 Unité Mixte de Recherche 7209 ‘Archéozoologie, Archéobotanique: Sociétés, Pratiques et Environnements’, Centre National de la Recherche Scientifique, Muséum national d’Histoire naturelle, Sorbonne Universités, CP 56, 55 rue Buffon, F-75005 Paris, France

## Abstract

Cremated human remains from Stonehenge provide direct evidence on the life of those few select individuals buried at this iconic Neolithic monument. The practice of cremation has, however, precluded the application of strontium isotope analysis of tooth enamel as the standard chemical approach to study their origin. New developments in strontium isotopic analysis of cremated bone reveal that at least 10 of the 25 cremated individuals analysed did not spend their lives on the Wessex chalk on which the monument is found. Combined with the archaeological evidence, we suggest that their most plausible origin lies in west Wales, the source of the bluestones erected in the early stage of the monument’s construction. These results emphasise the importance of inter-regional connections involving the movement of both materials and people in the construction and use of Stonehenge.

## Introduction

**Figure 1:**
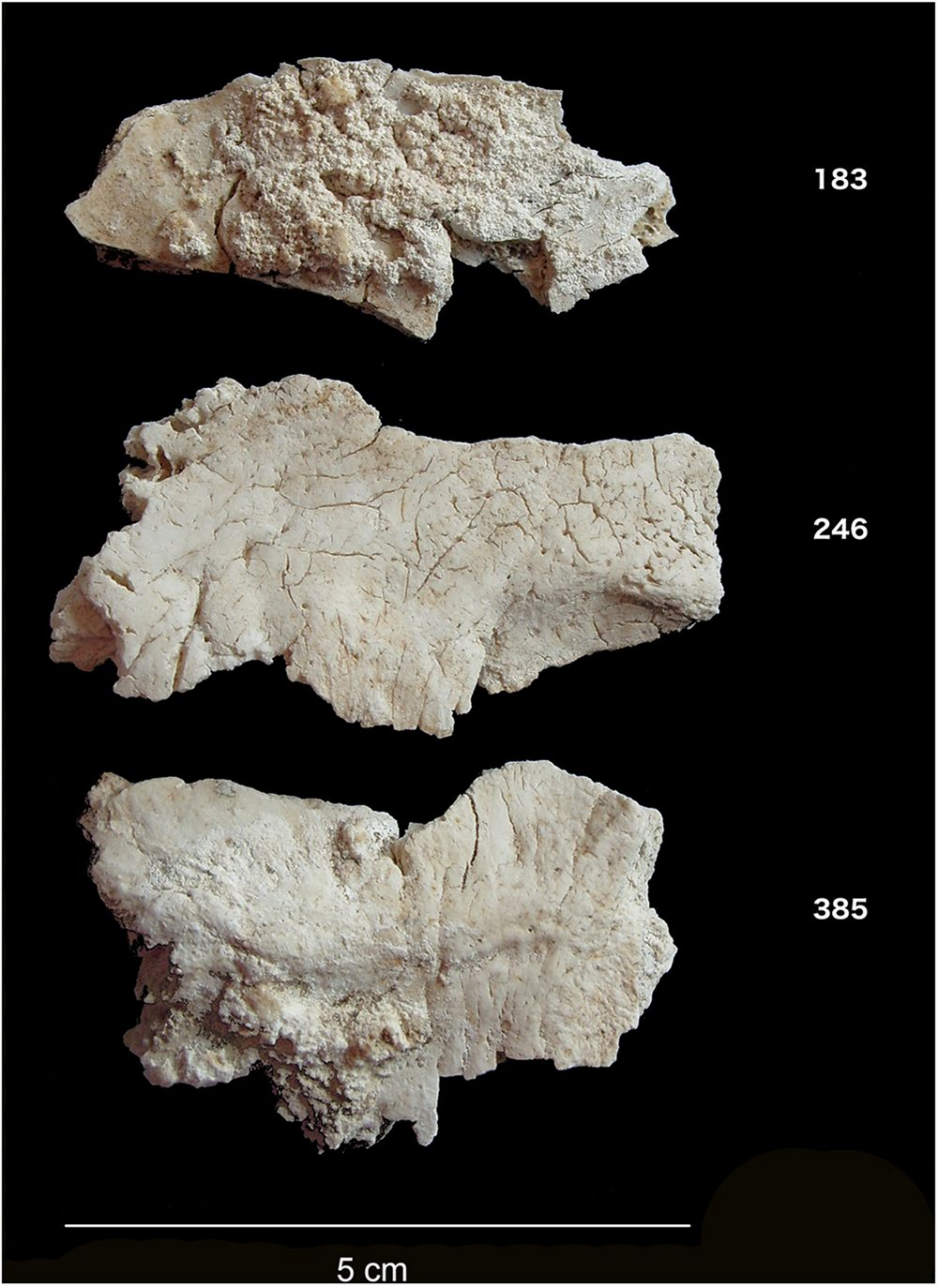
Cremated occipital bone fragments from Stonehenge.

Despite over a century of intense study of Stonehenge, we still know very little about the individuals buried at the site. Attention has focused rather on its monumental construction – the sourcing of the stones, their transport and construction, and on astronomical alignments. Stonehenge, however, also functioned as a cemetery from an early stage in its long history. Excavations in 1919–26 recovered the cremated remains of up to 58 individuals, making Stonehenge one of the largest Late Neolithic burial sites known in Britain (Fig. 1). Following their initial excavation, the cremated remains found in various ‘Aubrey Holes’ (a series of 56 pits placed around the inner circumference of the bank and ditch, named in honour of the seventeenth century antiquarian John Aubrey who first noted them) and elsewhere at the site were re-interred in Aubrey Hole 7 (AH7). This pit was re-excavated in 2008, and osteoarchaeological analysis identified central occipital bone fragments from at least 25 individuals. Direct radiocarbon dating places them in the centuries between 3180–2965 and 2565–2380 BC, reflecting the monument’s earlier stages of construction^1–3^, a period during which cremation was a common burial practice in Britain.

While the large sarsens (silicified sandstone) of the second stage of Stonehenge were most likely sourced ca. 20 kilometres north of the site, the bluestones (rhyolite, spotted dolerite and other lithologies) – now thought to have been erected in an earlier stage – have long been linked with the Preseli Hills of west Wales, over 200 km away, with some now more specifically sourced to Craig Rhos-y-felin and Carn Goedog quarries^4,5^. This raises questions about the nature of contacts between Wessex (south-central England) and western Britain, and the identity and origin of those chosen for burial at Stonehenge. Were they all drawn from communities in the immediate environs of Stonehenge, perhaps representing a local élite, albeit one possessing significant connections much further afield? Or did some people – as well as the stones – move here from elsewhere?

Unfortunately, cremation severely limits what can be learned about human remains from both traditional osteological and biogeochemical approaches. Isotopic studies of provenance usually focus exclusively on tooth enamel, as most resistant to diagenesis^6^, but cremation leads to enamel spalling and destruction. High temperatures also alter the stable carbon and oxygen isotope ratios of bone that might otherwise inform on diet and mobility (e.g.^7^) although they may still provide information on pyre characteristics such as temperature and ventilation^8–11^. Importantly for our purposes, fully calcined bone has recently been demonstrated to be a reliable substrate for preservation of the original strontium isotope (^87^Sr/^86^Sr) composition^12,13^, which reflects an average of the foods eaten over the last decade or so before death, in contrast to the childhood signal represented by dental enamel. In addition, Stonehenge lies on the Wessex chalk, characterized by a well-constrained range of strontium isotope ratios (±2 SD: 0.7074–0.7090) allowing for the identification of individuals consuming food beyond this landscape.

**Figure 2:**
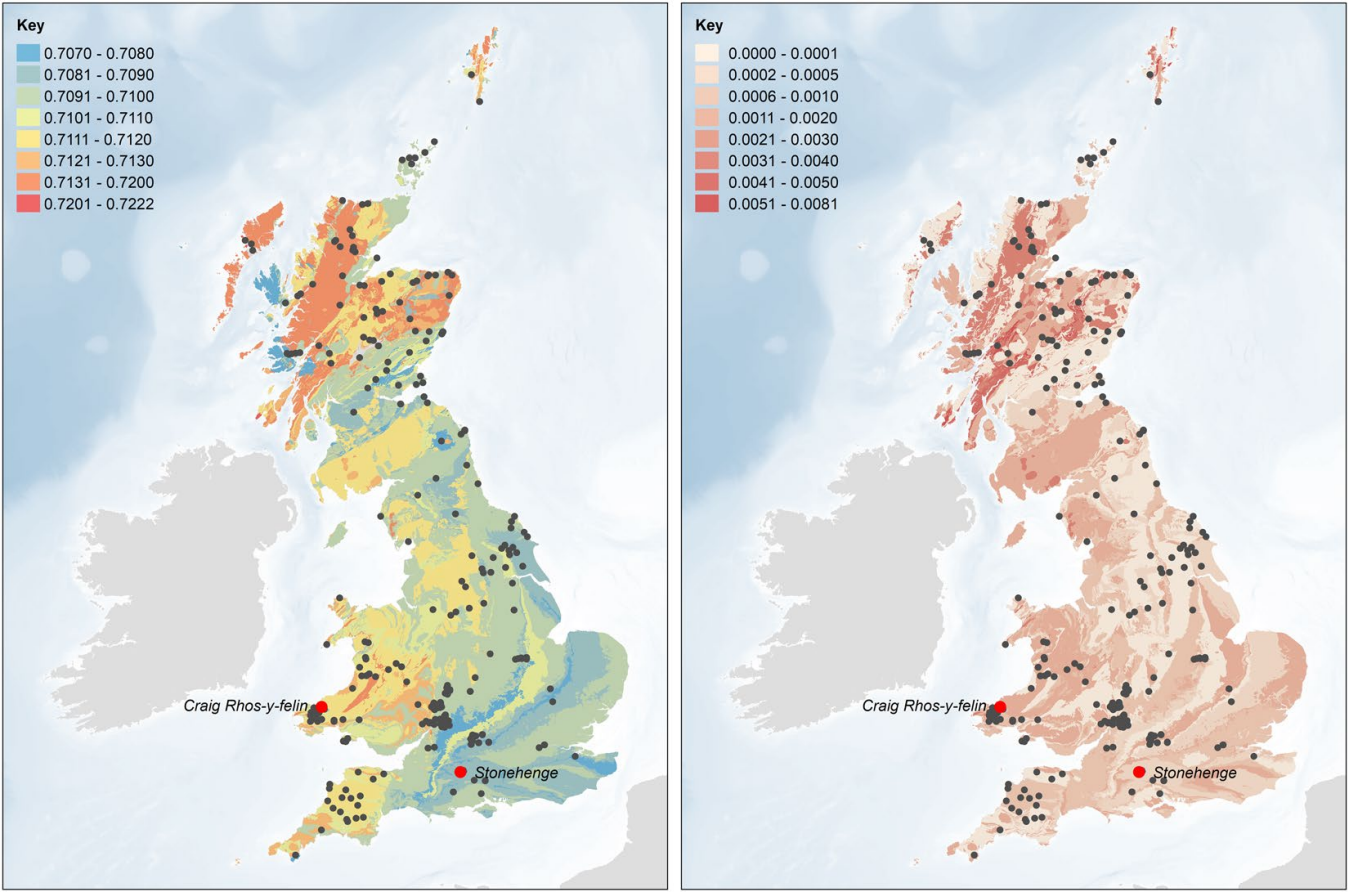
Biologically available strontium (BASr) baseline (left – mean and right – 1 SD), generated using the Spatial Join and Polygon to Raster tools in ArcGIS Desktop 10.6 (http://desktop.arcgis.com/en/arcmap/latest/tools/analysis-toolbox/spatial-join.htm and http://desktop.arcgis.com/en/arcmap/latest/tools/conversion-toolbox/polygon-to-raster.htm). Based upon BGS Geology 625k, with the permission of the British Geological Survey.

For this study, infrared, elemental and isotopic analyses were carried out on fragments of cremated occipital bone (Fig. 1) representing 25 distinct individuals at Stonehenge (Table S1). In addition, strontium isotope ratios (^87^Sr/^86^Sr) of 17 modern plant samples from eight locations in west Wales were measured (Table S2) and combined with previously published modern plant, water and dentine data from Britain^14,15^. This provides a baseline of the biologically available strontium (BASr) for Stonehenge and west Wales as well as for other parts of Britain (Fig. 2)^14–17^, which is important as the values for the underlying geological formations and soils do not necessarily translate directly into the biosphere^18^. The strontium isotope ratios for modern plants clearly distinguish the Ordovician and Silurian lithologies of west Wales (0.7095–0.7120) from the Cretaceous chalk of Wessex (0.7074–0.7090), which extends for at least 15 km around Stonehenge in all directions. Beyond this to the west and north is a large zone showing intermediate values (0.7090–0.7100)^14^ with small pockets of higher values^19^.

Previous strontium and oxygen isotopic research on human enamel concluded that the Beaker period (ca. 2400–1800 BC) ‘Boscombe Bowmen’ found near Stonehenge may have originated in west Wales^20^, or perhaps from even further afield, in Brittany^21^. Strontium isotope analysis has also been used on cattle from Durrington Walls, a large henge monument near to and contemporary with the later phases at Stonehenge (ca. 2500 BC), with some individual animals showing more radiogenic signals typical of the older bedrock of western or northern Britain^22^. None of this pre-supposes the outcome of the current study: both the Boscombe Bowmen and the Durrington Walls fauna post-date most of the cremations at Stonehenge by many centuries, and the movement of animals may have differed from that of people, especially with regard to the rights of certain individuals to be buried at Stonehenge^1^.

**Figure 3:**
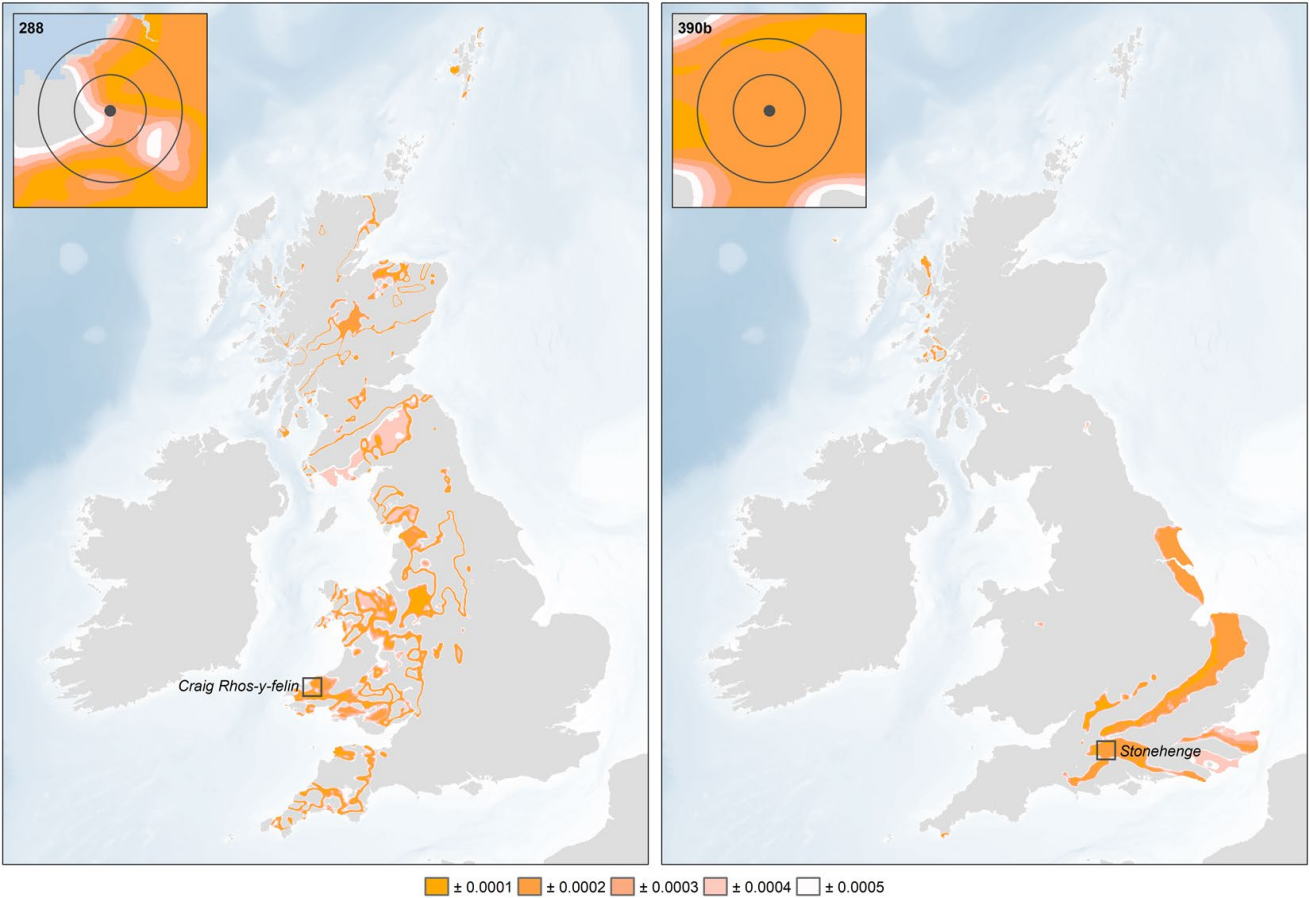
Geographic assignments of two of the sampled individuals (left – Sample 288, 0.7109, ‘non-local’; right – Sample 390b, 0.7079, ‘local’) based on the residuals between the measured ^87^Sr/^86^Sr isotope ratio and the focal mean of the BASr baseline (5 km search radius), calculated using the Focal Statistics and Raster Calculator tools in ArcGIS 10.6 (http://desktop.arcgis.com/en/arcmap/latest/tools/spatial-analyst-toolbox/focal-statistics.htm and http://desktop.arcgis.com/en/arcmap/latest/tools/spatial-analyst-toolbox/raster-calculator.htm).

**Figure 4:**
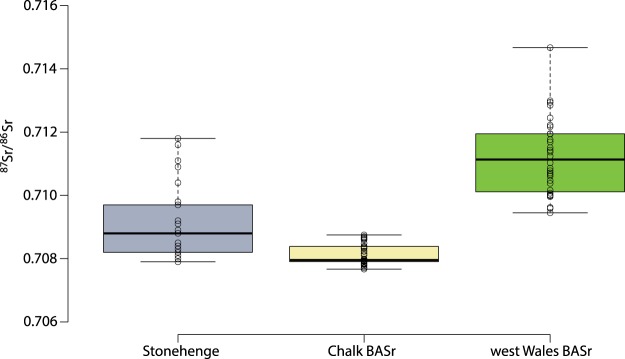
^87^Sr/^86^Sr results for cremated human remains from Stonehenge and biologically available strontium values (BASr) from the Wessex chalk and west Wales.

The infrared spectroscopy results confirm that all samples were fully calcined^10,11^. The ^87^Sr/^86^Sr ratios for the cremated human remains from Stonehenge range from 0.7078 to 0.7118 (Figs 3 and 4). There is no consistent relationship between the strontium isotope results and the radiocarbon dates. We consider the fifteen individuals with strontium isotope ratios falling below 0.7090 as ‘local’ inasmuch as they clearly reflect the chalk geology, although it must be acknowledged that this extends for at least 15 km in all directions, and further in some (see Fig. 2). With values ranging from 0.7091 to 0.7118, the remaining ten individuals (40%) could not have consumed food growing around Stonehenge alone (Fig. 2) for the last ten or so years of their lives. Those with the highest values (>0.7110) point to a region with considerably older and more radiogenic lithologies, which would include parts of southwest England (Devon) and Wales (parsimony making locations further afield – including parts of Scotland, Ireland and continental Europe – less probable). Those ‘non-locals’ with intermediate values could reflect places closer to Stonehenge or a mixture of different sources (e.g. chalk or other limestones and more radiogenic lithologies). Since measurements on bone reflect a mixture of the foods consumed over the decade or so prior to death, there is also a temporal aspect to be considered. For example, those moving later in life from west Wales to the vicinity of Stonehenge would present a signal increasingly attenuated by the consumption of local foods, while migrants arriving on the Wessex chalk more than a decade before death would effectively become ‘local’ in terms of their bone strontium isotope ratio. Complex patterns of movements in both directions are possible, with individuals originating in Wessex moving to west Wales, and incorporating a higher, more radiogenic ^87^Sr/^86^Sr signal. Obviously, any such individuals would only feature in the present study if they subsequently returned to Stonehenge either before death, or afterwards in the form of cremated remains.

Further infrared data show that only four samples contain cyanamide (–CN_2_H) suggesting that the cremations took place under oxidizing conditions, i.e., high oxygen-to-fuel ratio, as would be produced in small and/or well-ventilated pyres^10^. The carbon (δ^13^C) and oxygen (δ^18^O) isotope ratios of the carbonate fraction of the cremated bone apatite show a broad range of un-correlated values in contrast to observations from Neolithic sites in Ireland where correlations were observed^11^, suggesting that the Stonehenge individuals were cremated under more variable conditions (e.g. pyre settings, amount, type and origin of the wood used as fuel).

**Figure 5:**
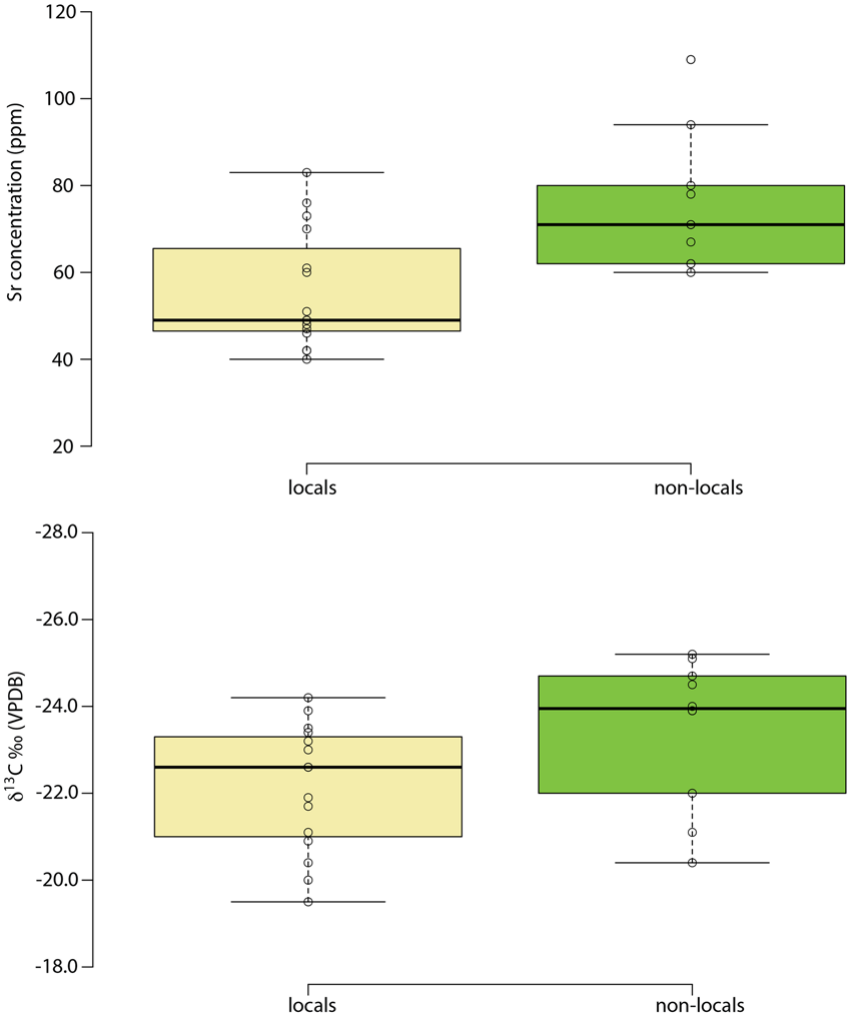
Differences between ‘locals’ and ‘non-locals’ in (**a**) strontium concentration and (**b**) δ^13^C.

The ‘locals’ as identified by strontium isotope ratios also exhibit a lower elemental strontium concentration (Student’s t-test, *p* = 0.003; Cohen’s *d* = 1.35) (Fig. 5a). The interpretation of this difference, however, is not straightforward. Chalk would be expected to have a higher Sr concentration than the varied lithologies of west Wales, but this needs to be balanced against the ratio of Sr to calcium (Ca), since Sr substitutes for Ca in the skeleton, and this ratio is higher in the mudstones and siltstones that characterise west Wales than in carbonate rock^23^. Another possibility is that there was a dietary difference between the two regions, with greater reliance on plant foods in west Wales compared to Wessex (biopurification causing a sharp drop in Sr concentration between plants and animal flesh/milk), though admittedly this is hard to envisage given that both regions are primarily suited to pasture. Further research is required to explain the observed difference. The ‘locals’ also have higher δ^13^C values compared to the ‘non-locals’ (Mann-Whitney U-test, *p* = 0.010; Spearman’s *r* = 0.535) (Fig. 5b). Given the relative isotopic homogeneity of Neolithic diets in Britain (based on a C_3_ terrestrial system), this is unlikely to reflect dietary differences^24^. This is particularly so since the δ^13^C of cremated bone largely reflects the values of the fuel used for the cremation pyres, with between 40 and 95% of the wood values being incorporated into the cremated bone signal^25,26^. This in turn is related to the trees’ growing conditions, especially as regards the amount of light received^27^. Thus, the higher δ^13^C values seen in the ‘locals’ suggest the use of pyre wood grown in a relatively open landscape, consistent with conditions on Wessex’s chalk downlands^28^. The lower values, by contrast, suggest wood fuel taken from comparatively dense woodland, such as would have been found in Wales^29^. Together, the infrared and carbon isotope results suggest that the cremations of those buried at Stonehenge took place under different conditions, using different types of fuel. Moreover, the link between non-locals and lower δ^13^C values suggests that some individuals may have been cremated in west Wales and their remains subsequently brought to Stonehenge. This recalls Hawley’s observation during his 1920s excavations that the cremated remains in the Aubrey Holes appeared to have been deposited in organic containers such as leather bags, leading him to suggest that they “had apparently been brought from a distant place for interment” (1928: 158)^30^.

We conclude that at least 40% of those buried at Stonehenge did not exclusively spend the last decade or so of their lives in the environs of the site, or indeed anywhere on the chalklands of southern England. The highest strontium isotope ratios are consistent with living on geological formations in western Britain, a region that includes west Wales, the known source of Stonehenge’s bluestones. While strontium isotope ratios on their own cannot distinguish between places with similar values, this connection suggests that west Wales is the most likely origin of at least some of these individuals. Indeed, all the measurements fall between the biologically available strontium values for Stonehenge and west Wales, consistent with people moving between the two locations at different times in their lives. Finally, the results suggest that at least some ‘non-local’ individuals were cremated away from Stonehenge, and that their cremated remains were brought to the site for burial, perhaps in conjunction with the raising of the bluestones. This is particularly compelling in light of the recent suggestion that the bluestones originally stood in the Aubrey Holes in which most of the cremations were found^1^.

## Materials and Methods

### Samples

A total of 31 cremated bone samples from 25 individuals were analyzed, all of which have been previously radiocarbon dated^3^. Of the 25 individuals, 24 came from the excavation of the Aubrey Hole 7 where they had been re-buried in 1935. The last individual, a woman, was found just next to Aubrey Hole 7. Three samples represent juveniles while the remainder are adults. While it is difficult to sex cremated remains, it was still possible to identify six possible females and nine possible males, with the remainder unsexed^3^. In addition, 17 modern plant samples from west Wales were analysed to better characterize the biologically available strontium around the quarries where bluestones originate.

### Infrared analyses

The cremated bone samples were first crushed using a mortar and pestle and then analysed by FTIR-ATR (Agilent Technologies Cary 640 FTIR with GladiATR^TM^ from Pike Technologies). Each sample was pressed onto the diamond crystal and measured in triplicate (on different aliquots if sufficient material was available). The background was measured and removed and a baseline correction was carried out using Agilent Resolution Pro. The spectra were normalised and averaged before calculation of a suite of infrared indices that describe the composition and structure of the bone^10^.

Various infrared indices were calculated for each sample (Table S1 – see 8, 10 for more details). The cyanamide to phosphate ratio (CN/P) is of particular interest as it characterizes the presence of cyanamide, which has been suggested to be present when bone is burned in reducing conditions^10,11^. The IRSF has been shown to be temperature dependent while the BPI characterizes the amount of remaining carbonates present in bone after cremation.

### Carbon and oxygen stable isotope ratios

Before isotopic analysis, the calcined bone fragments were treated with sodium hypochlorite (1% NaOCl) for one hour to remove any remaining organic matter followed by three rinses with MilliQ water and then a four-hour treatment with 1 M acetic acid (CH_3_COOH) to remove any adsorbed carbonates and diagenetic apatites. The samples were then rinsed three times with MilliQ water and left in the freeze-dryer overnight. Between 3 and 5 mg were reacted with 100% phosphoric acid at 70 °C for 4 min in a Kiel IV autocarbonate device interfaced with a Delta V Advantage isotope ratio mass spectrometer at the SSMIM (Service de Spectrométrie de Masse Isotopique du Muséum National d’Histoire Naturelle), Paris, France. A laboratory carbonate standard (LM marble) normalized with NBS 19 and giving mean δ^13^C and δ^18^O values of +2.08 ± 0.04‰ and +1.70 ± 0.05‰, respectively, (n = 78) was used to check the accuracy and precision of the data. We used these standard deviations as an indicator of analytical precision over the period of analysis. The carbon and oxygen isotope ratios are, by convention, expressed relative to a standard (VPDB – Vienna Pee Dee Belemnite). Both are expressed in per mil (‰ – 1/1000):

$$\begin{array}{c}{\delta }^{13}C=\frac{{({}^{13}C/{}^{12}C)}_{sample}-{({}^{13}C/{}^{12}C)}_{vPDB}}{{({}^{13}C/{}^{12}C)}_{vPDB}}\times 1000\,\& \\ {\delta }^{18}O=\frac{{({}^{18}O/{}^{16}O)}_{sample}-{({}^{18}O/{}^{16}O)}_{vPDB}}{{({}^{18}O/{}^{16}O)}_{vPDB}}\times 1000\end{array}$$

### Strontium isotope analysis

Two isotopes of strontium, ^86^Sr and ^87^Sr, are widely used in mobility studies of humans and fauna. Strontium-87 is the product of the radioactive decay of rubidium-87 (^87^Rb), so strontium isotope ratios (^87^Sr/^86^Sr) vary between different types of bedrock, depending on the initial ^87^Sr/^86^Sr ratio, the Rb/Sr ratio and the age. The older and more Rb-enriched the bedrock, the more enriched it is in ^87^Sr^31^. Soluble strontium is then taken up by plants and enters the bones and teeth of humans and animals by replacing calcium in the bioapatite fraction of bone and teeth. Hence, strontium isotope ratios (^87^Sr/^86^Sr) can be measured on bone and teeth to suggest places of origin for animals and humans. The residence time of strontium in bone apatite is not fully known but here we assume that it will be around 10 years.

Strontium isotope ratios were measured by Multi-Collector Inductively-Coupled-Plasma Mass-Spectrometry (MC-ICP-MS) following the procedure detailed in^12^. Cremated bones were pretreated with 1 M acetic acid (1 mL per 10 mg of sample) for 3 min in an ultrasonic bath, followed by three rinses with MilliQ water and 10 min ultrasonication. Plant samples were simply ashed in a muffle furnace at 650 °C. The entire acid digestion process and subsequent Sr purification were achieved under a class 100 laminar flow hood in a class 1000 clean room (Université Libre de Bruxelles, Belgium, hereafter ULB). Fifty mg of sample were digested in subboiled concentrated HNO_3_ at 120 °C for 24 h, before purification of the Sr analyte by a chromatographic technique on ion-exchange resins (see 12 for more details). The isotope ratios of the purified strontium samples were then measured on a Nu Plasma MC-ICP mass spectrometer (from Nu Instruments) at ULB. During the course of this study, repeated measurements of the NBS987 standard solution yielded ^87^Sr/^86^Sr = 0.710214 ± 40 (2 SD for 15 analyses), which is, for our purposes, largely sufficiently consistent with the average value of 0.710252 ± 13 (n = 88) obtained by TIMS (Thermal Ionization Mass Spectrometry)^32^. All the data were corrected for mass fractionation by internal normalization to ^86^Sr/^88^Sr = 0.1194. In addition, after the measurements all the rough data were normalised using a standard-sample bracketing method with the recommended value of ^87^Sr/^86^Sr = 0.710248^32^. For each sample the ^87^Sr/^86^Sr value is reported with a 2σ error (absolute error value of the individual sample analysis − internal error).

### Strontium concentrations

Small sample fractions (~1 to 3 mg) pre-treated as above were digested in precleaned Teflon beakers (Savillex) using subboiled 7 M HNO_3_ at 120 °C for 24 h, evaporated to near-dryness and subsequently digested with a drop of concentrated HNO_3_. Following dilution with 2% HNO_3_, Sr and Ca concentrations (ppm) in the sample digests were determined using a Thermo Scientific Element 2 sector field ICP mass spectrometer at the Vrije Universiteit Brussel (VUB, Belgium) in low (^86^Sr and ^88^Sr) and medium (^43^Ca and ^44^Ca) resolution using Indium (In) as an internal standard and external calibration versus a calibration curve. Accuracy was evaluated by the simultaneous analysis of limestone reference material SRM8544 (NBS19) and comparison to available published literature data^33,34^. Based on repeated digestion and measurement of this reference material, the analytical precision (1 SD) of the procedure outlined above is estimated to be better than 3%.

### Statistical analysis

Data were assessed for normality using Shapiro-Wilk tests and then analysed with parametric (Student’s t) or non-parametric (Mann-Whitney U) tests as appropriate. For distributions not departing significantly from normality, Cohen’s *d* was used to assess effect size, a measure of the strength of a difference between two sample means, beyond its simple existence. By convention, *d* values of less than 0.2 (the difference in sample means expressed in pooled standard deviations) are taken to be small (i.e., meaningless regardless of the *p* value), while those above 0.7 are considered large^35^. For non-normal distributions, the strength of the relationship between the two variables was assessed using Spearman’s correlation coefficient *rho*^36^.

### Map of the biologically available strontium

An updated version of the map of biosphere strontium isotope variation across Britain (Fig. 1b in^14^) was generated using British Geological Survey 1:625,000 bedrock geology digital mapping (DiGMapGB-625 – Figure S1) and additional strontium data from key regions in southern Britain. The dataset from Evans *et al*. 2010 was supplemented with data for plants growing on key rock types in south-west Wales (Table S2), the Gower peninsula (Schulting *et al*. n.d.), Gloucester^19^ and the Ridgeway^15^. Mean values and standard deviations were calculated for samples corresponding to each polygon in the DiGMapGB-625 dataset. Where possible, only values from plant samples were used to calculate the mean values and standard deviations for each polygon. Where no plant data were available, mean values and standard deviations were calculated, in order of preference, as follows:

1. Other samples – using values from bone, dentine, soil and water samples;
2. Rock types – using values from other polygons of the same rock type;
3. Isotope packages – using values for other polygons of the same isotope package.

Outliers, identified as those measurements lying more than 3 IQR (interquartile range) in their isotope package, were removed from the analysis (Figures S2 and S3). The bedrock polygons were converted to a raster dataset with a cell size of 100 m (to ensure that small polygons were retained, and cell values corresponding to the mean value of the dominant rock type (the rock type with the largest combined area)). A baseline of biologically available strontium was created by calculating the focal mean of each cell in the raster dataset, i.e. the mean value of all cells within a 5 km search radius, using an extension of the methodology employed for the analysis of strontium data from Neolithic and Bronze Age sites in Northern Ireland^17^. Continuous surfaces showing the absolute deviation from the focal mean were subsequently generated for each of the individuals from Stonehenge, with cells with the lowest values representing locations with focal means that are similar to the measured values, i.e. the locations from which the individuals are most likely to have originated (Figures S4, S5 and S6).

### Defining ‘local’

In the absence of wheeled transport, small-scale, intensive agriculture being proposed for the Neolithic and Bronze Age tends to focus on fields within 1 km of settlements^37,38^. Of course use would also be made of more distant fields, but since the Cretaceous chalk extends for at last 15 km in all directions around Stonehenge its strontium isotope range should provide a robust definition of ‘locals’. Those individuals making repeated use of fields with significantly higher ^87^Sr/^86^Sr values to the west or north would be expected to exhibit higher values in their bone, which presents an average of bioavailable strontium in foods consumed over the last decade or so of life. Given the mobility of cattle and sheep, some pastures may also have been at greater distances from settlements, but meat and milk contain relatively little strontium compared to plants, and so the contribution made by livestock would be heavily biased against. As such, using the updated version of the map of the biologically available strontium for the UK it is possible to define ‘local’ and ‘non-local’ individuals. ‘Locals’ are characterized by values below 0.7090 (Fig. 4). The origin of the ‘non-locals’ was evaluated using the continuous surfaces created for each individual (see above and Figs 3, S4, S5 and S6).

## Electronic supplementary material

Supplementary Material
