# Supplementary material for: Strontium isotope analysis on cremated human remains from Stonehenge support links with west Wales
Source: Sci Rep. 2018 Aug 2;8:10790. doi: 10.1038/s41598-018-28969-8 (PMC6072783; doi:10.1038/s41598-018-28969-8)

SUPPLEMENTARY MATERIALS

Strontium isotope analysis on cremated human remains from Stonehenge support links with west Wales

Christophe Snoeck[1,2,*], John Pouncett[1], Philippe Claeys[2], Steven Goderis[2], Nadine Mattielli[3], Mike Parker Pearson[4], Christie Willis[4], Antoine Zazzo[5], Julia A. Lee-Thorp[1], Rick J. Schulting[1]

[1]School of Archaeology, University of Oxford, Dyson Perrins Building, South Parks Road, Oxford, OX1 3QY, UK

[2]Research Unit: Analytical, Environmental & Geo-Chemistry, Dept. of Chemistry, Vrije Universiteit Brussel, AMGC-WE-VUB, Pleinlaan 2, 1050 Brussels, Belgium

[3]G-Time Laboratory, Université Libre de Bruxelles, CP 160/02, 50, Avenue F.D. Roosevelt, B-1050 Brussels, Belgium

[4]Institute of Archaeology, 31-34 Gordon Square, University College London, WC1H 0PY, UK

[5]Unité Mixte de Recherche 7209 'Archéozoologie, Archéobotanique : Sociétés, Pratiques et Environnements', Centre National de la Recherche Scientifique, Muséum national d'Histoire naturelle, Sorbonne Universités, CP 56, 55 rue Buffon, F-75005 Paris, France

*Correspondence to: christophe.snoeck@vub.be

SUPPLEMENTARY TABLES

Table S1 – Infrared results, isotope ratios and Sr concentrations for cremated human bone from Stonehenge

Lab no.	Sample ref.	^{14}C Lab-code	Date** (Uncal. BP)	Date*** (Cal. BC)	BPI*	IRSF *	CN/P*	$\delta^{13}C$ (‰)	$\delta^{18}O$ (‰)	$^{87}Sr/^{86}Sr$	± 2σ	[Sr] (ppm)
St24	7	OxA-27086	4315 ± 35	3020-2885	0.64	4.80	0.14	-25.1	-17.1	0.709699	0.000014	67
St01	110	OxA-26962	4280 ± 20	2915-2885	0.26	4.41	0.20	-21.7	-18.5	0.708169	0.000011	49
St02	173	OxA-26963	4360 ± 35	3090-2900	0.33	4.50	0.20	-23.9	-16.3	0.708334	0.000011	70
St22	211	OxA-27085	4340 ± 30	3025-2895	0.37	5.03	0.37	-23.5	-18.5	0.708375	0.000009	42
St03	221	OxA-26964	4325 ± 30	3015-2890	0.49	4.49	0.12	-24.0	-17.0	0.709211	0.000011	71
St04	223	OxA-26965	4100 ± 30	2865-2500	0.34	4.83	0.18	-22.6	-14.8	0.708444	0.000011	48
St05	225	OxA-27089	4130 ± 30	2870-2585	0.24	4.63	0.16	-22.2	-13.7	0.708439	0.000012	50
St28		GU-28587	4220 ± 20	2900-2710	0.28	4.59	0.18	-21.6	-14.7	0.708613	0.000007	52
Av					0.26	4.61	0.17	-21.9	-14.2	0.7085	/	51
St06	227	OxA-26966	4170 ± 30	2880-2635	0.46	4.41	0.12	-24.6	-15.8	0.708093	0.000011	73
St29		GU-28588	4105 ± 20	2855-2580	0.43	4.37	0.10	-23.8	-18.1	0.708111	0.000017	79
Av					0.44	4.39	0.11	-24.2	-17.0	0.7081	/	76
St07	246	OxA-27045	4455 ± 35	3340-2945	0.31	4.42	0.23	-21.1	-14.1	0.709784	0.000010	62
St08	255	OxA-27046	4195 ± 30	2890-2675	0.87	4.08	0.20	-19.2	-17.7	0.708093	0.000011	71
St30		GU-28589	4165 ± 20	2880-2670	0.71	3.90	0.21	-20.8	-17.4	0.708058	0.000013	74
Av					0.79	3.99	0.21	-20	-17.6	0.7081	/	73
St09	280	OxA-27047	4375 ± 30	3090-2910	0.27	4.21	0.16	-20.9	-16.0	0.708001	0.000012	47
St10	281	OxA-27048	4210 ± 30	2900-2680	0.30	4.78	0.22	-24.7	-15.2	0.709092	0.000012	80
St11	288	OxA-27049	4235 ± 30	2910-2700	0.35	4.67	0.18	-23.9	-17.6	0.710858	0.000010	109
St25	289	OxA-30294	4390 ± 30	3090-2920	0.30	4.33	0.24	-21.1	-19.9	0.708207	0.000010	40
St12	307	OxA-27077	4420 ± 30	3320-2920	0.40	4.71	0.10	/	/	/	/	/
St27		GU-28586	4385 ± 20	3090-2920	0.69	4.94	0.17	-24.5	-16.2	0.710352	0.000013	71
Av					0.55	4.83	0.14	/	/	/	/	/
St13	330	OxA-27078	4255 ± 35	2920-2700	0.32	4.75	0.11	-25.2	-17.8	0.709710	0.000012	60
St14	334	OxA-27079	4390 ± 30	3090-2920	0.19	4.32	0.17	/	/	/	/	/
St26		GU-28585	4395 ± 20	3090-2925	0.24	3.95	0.22	-22.6	-17.6	0.708903	0.000014	83
Av					0.22	4.14	0.20	/	/	/	/	/
St15	336	OxA-27090	4415 ± 30	3315-2920	0.29	4.42	0.16	-23.9	-19.5	0.711607	0.000012	78
St21	344	OxA-27092	4425 ± 20	3310-2930	0.44	4.72	0.13	-23.2	-21.7	0.708755	0.000009	42
St16	357	OxA-27080	4325 ± 30	3020-2890	0.26	4.56	0.19	-22.5	-16.4	0.711136	0.000010	54
St31		GU-28590	4350 ± 20	3020-2910	0.22	4.42	0.38	-21.5	-17.3	0.710974	0.000015	69
Av					0.24	4.49	0.29	-22.0	-16.9	0.7111	/	62
St17	366	OxA-27081	4350 ± 30	3080-2900	0.24	4.73	0.28	-23.0	-17.0	0.708103	0.000008	49
St18	389	OxA-27082	4405 ± 25	3095-2920	0.23	4.18	0.22	-19.5	-18.3	0.708890	0.000012	61
St19	390b	OxA-27083	4260 ± 20	2910-2880	0.24	4.64	0.42	-20.4	-18.0	0.707854	0.000012	60
St20	596	OxA-27084	4365 ± 30	3085-2905	0.31	4.75	0.22	-20.4	-19.1	0.711767	0.000010	94
St23	382+323	OxA-27093	4180 ± 35	2890-2635	0.17	4.86	0.21	-23.4	-17.9	0.708251	0.000012	46

*Data from 10

** Data from 3

*** Calibrated radiocarbon dates (95% confidence interval) using OxCal v4.2.[39]

BPI: type B carbonate/phosphate index

IRSF: Infrared Splitting Factor ('crystallinity')

CN/P: cyanamide/phosphate ratio

Table S2 – Strontium isotope ratios and GPS location of modern plant samples

	NORTH	WEST	$^{87}Sr/^{86}Sr$	2σ
WW1 grass	51,831590	-4,7343307	0.711686	0.000011
WW1 tree 1	51,831590	-4,7343307	0.711947	0.000010
WW1 tree 2	51,831590	-4,7343307	0.712846	0.000010
WW2 grass	51,992310	-4,7438580	0.712935	0.000008
WW2 quarry	51,992310	-4,7438580	0.710465	0.000007
WW2 shrub	51,992310	-4,7438580	0.711447	0.000020
WW3 shrub	51,999077	-4,7693553	0.709590	0.000009
WW4 grass	51,976147	-4,7005525	0.711239	0.000010
WW4 shrub	51,976147	-4,7005525	0.709618	0.000010
WW5 grass	51,982020	-4,6880784	0.707953	0.000008
WW5 tree	51,982020	-4,6880784	0.708394	0.000008
WW6 grass	51,824920	-4,4743443	0.712216	0.000011
WW6 shrub	51,824920	-4,4743443	0.710861	0.000009
WW7 grass 1	51,810497	-4,4726890	0.711177	0.000009
WW7 grass 2	51,810497	-4,4726890	0.711373	0.000017
WW7 shrub	51,810497	-4,4726890	0.710706	0.000014
WW8 tree	51,839985	-4,2871723	0.710687	0.000007

Additional reference

39. Bronk Ramsey, C. OxCal 4.2. Web Interface Build (78) In: https://c14.arch.ox.ac.uk/ (2013)

SUPPLEMENTARY FIGURES

FIGURE S1 – Bedrock geology, produced using ArcGIS Desktop 10.6. Based upon BGS Geology 625k, with the permission of the British Geological Survey.

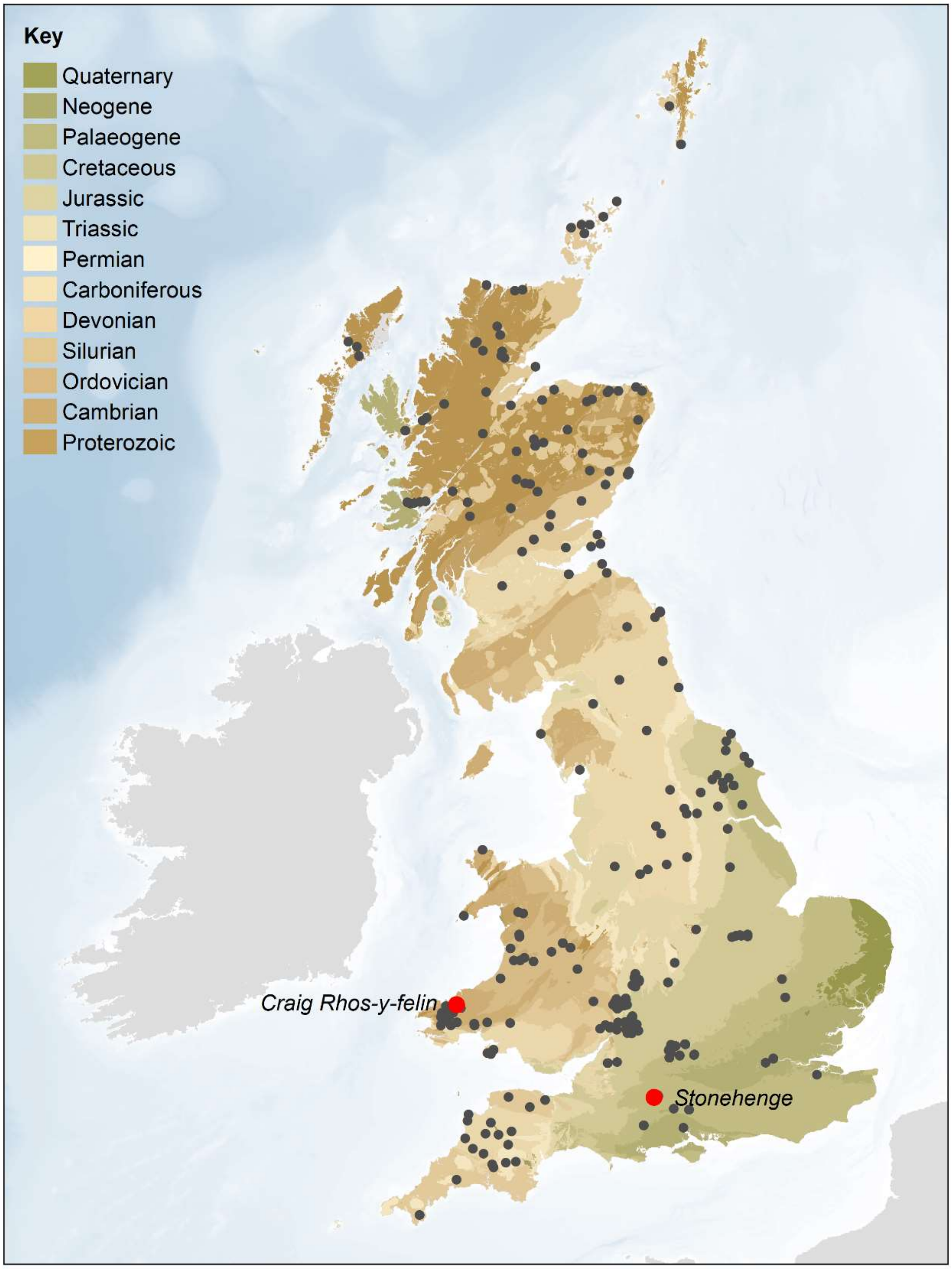

FIGURE S2 – $^{87}Sr/^{86}Sr$ values of the plant, bone/dentine, enamel and water samples used to generate the BASr baseline, grouped by isotope 'package' (14, Figure 1b), produced using Tableau Desktop 10.5. Based on BGS Geology 625k, with the permission of the British Geological Survey.

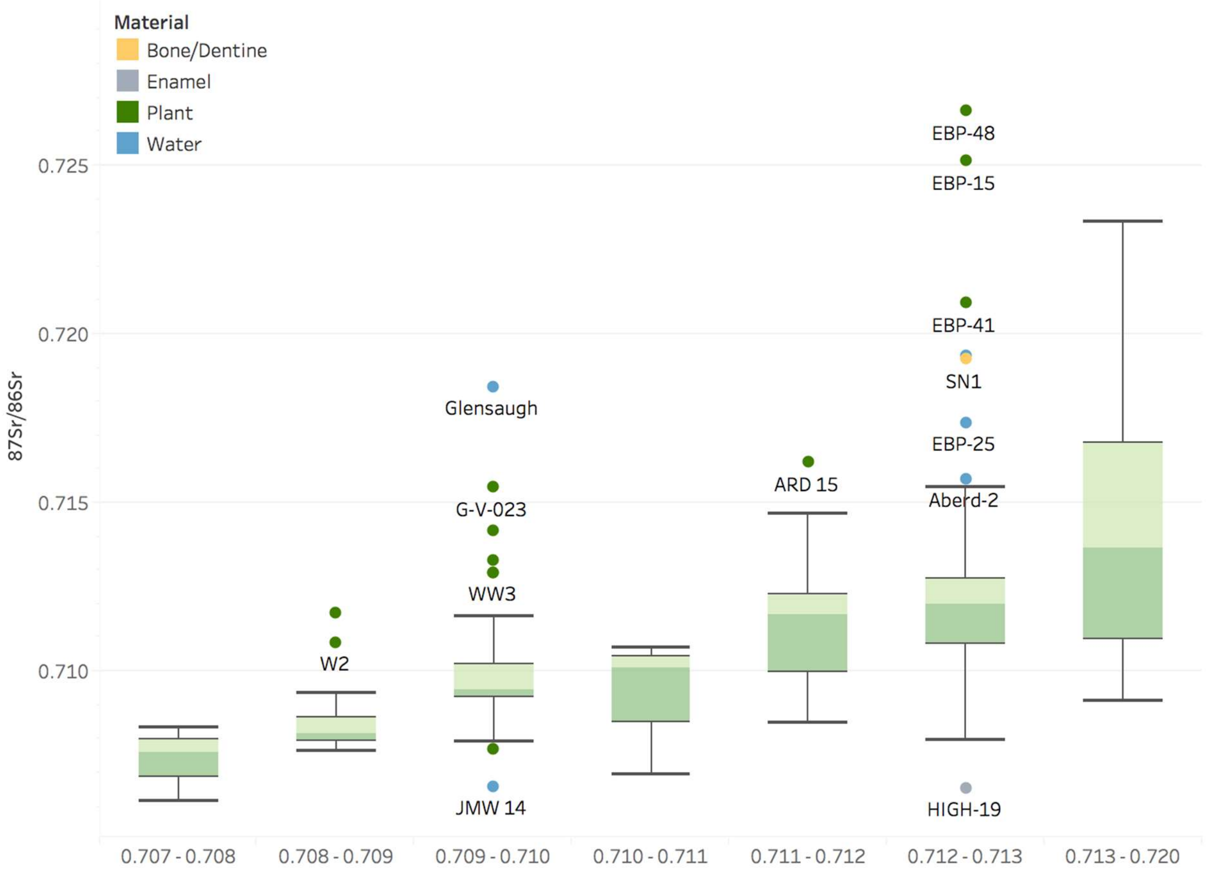

FIGURE S3 – Groups of samples used to generate the BASr baseline, produced using ArcGIS Desktop 10.6. Based upon BGS Geology 625k, with the permission of the British Geological Survey.

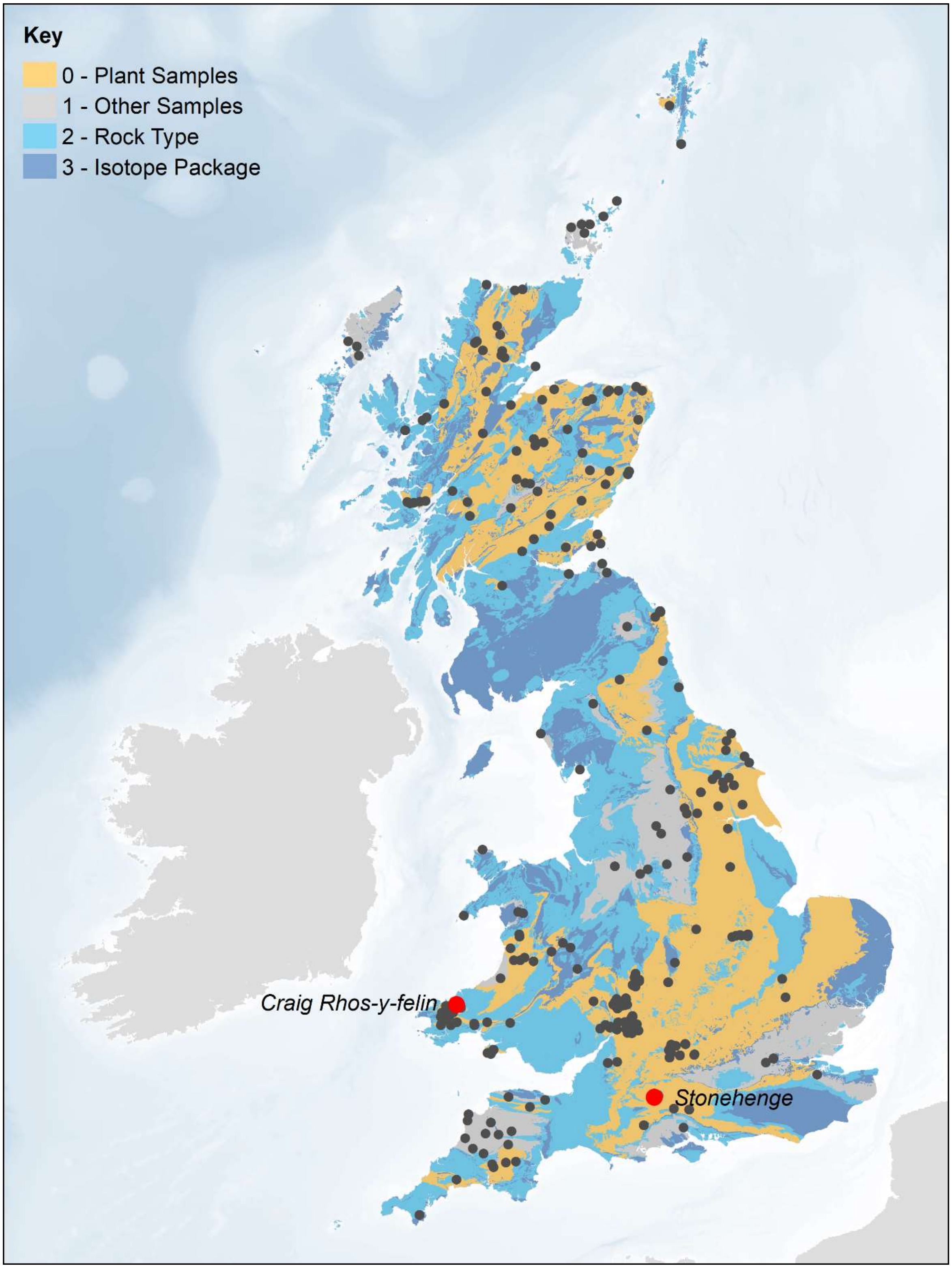

FIGURE S4 – Geographic assignments of the sampled individuals based on the residuals between the measured $^{87}Sr/^{86}Sr$ isotope ratio and the focal mean of the BASr baseline (5km search radius), calculated using the Focal Statistics and Raster Calculator tools in ArcGIS 10.6 (http://desktop.arcgis.com/en/arcmap/latest/tools/spatial-analyst-toolbox/focal-statistics.htm and http://desktop.arcgis.com/en/arcmap/latest/tools/spatial-analyst-toolbox/raster-calculator.htm).

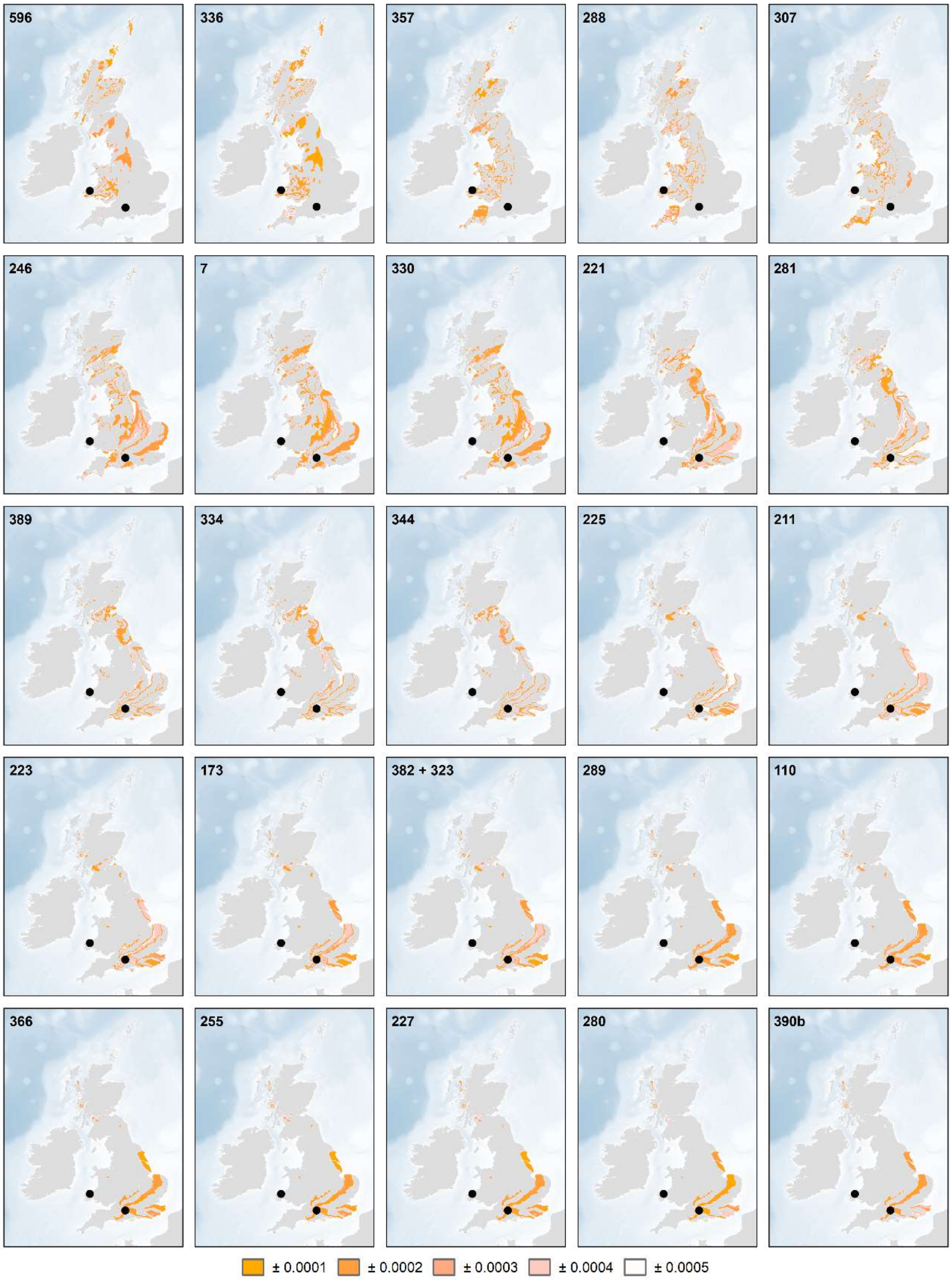

FIGURE S5 – Geographic assignments of the sampled individuals based on the residuals between the measured $^{87}Sr/^{86}Sr$ isotope ratio and the focal mean of the BASr baseline (5km search radius), centred on 5km and 10km BASr catchments for Craig Rhos-y-Felin, calculated using the Focal Statistics and Raster Calculator tools in ArcGIS 10.6 (http://desktop.arcgis.com/en/arcmap/latest/tools/spatial-analyst-toolbox/focal-statistics.htm and http://desktop.arcgis.com/en/arcmap/latest/tools/spatial-analyst-toolbox/raster-calculator.htm).

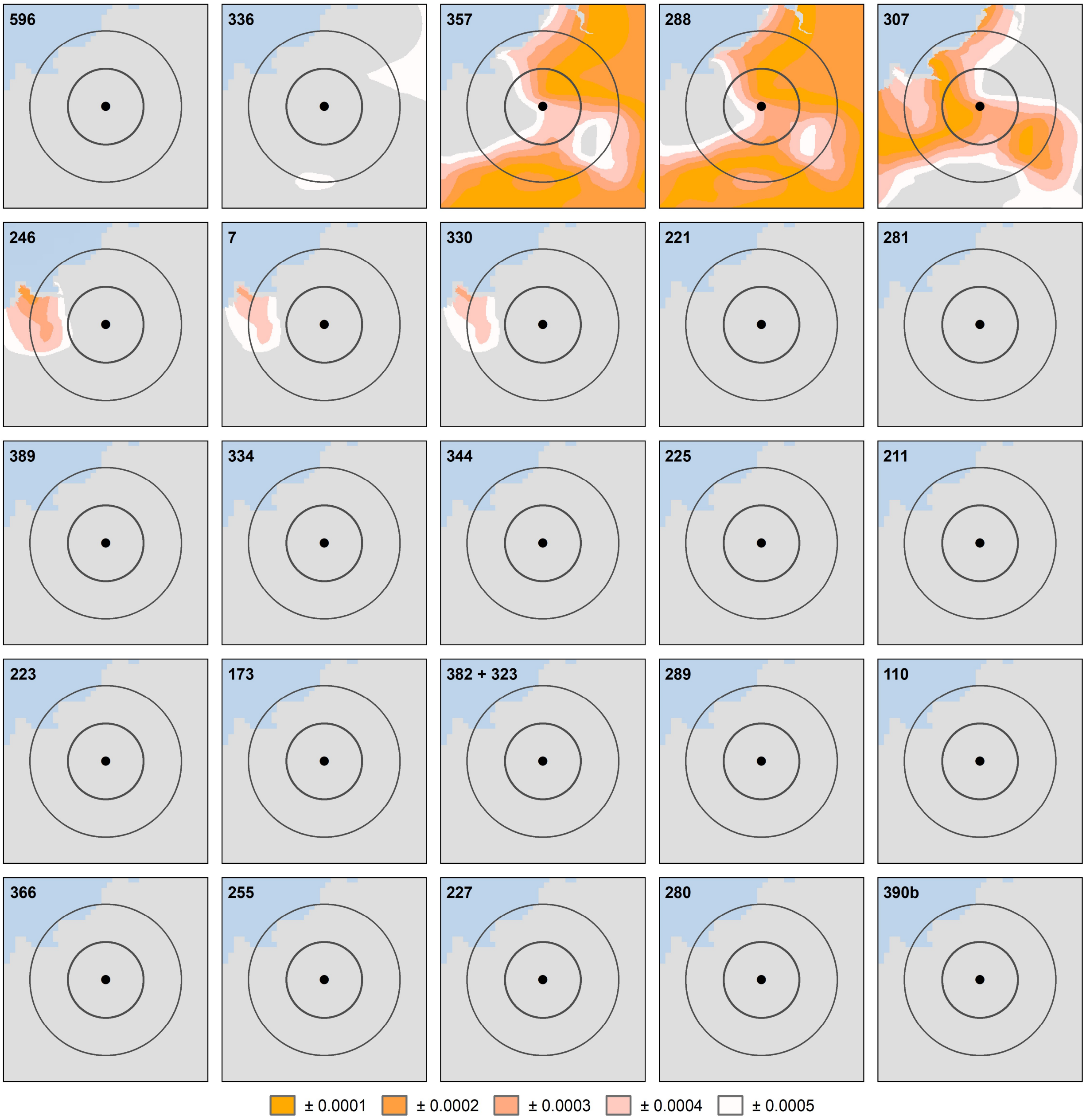

FIGURE S6 – Geographic assignments of the sampled individuals based on the residuals between the measured $^{87}Sr/^{86}Sr$ isotope ratio and the focal mean of the BASr baseline (5km search radius), centred on 5km and 10km BASr catchments for Stonehenge, calculated using the Focal Statistics and Raster Calculator tools in ArcGIS 10.6 (http://desktop.arcgis.com/en/arcmap/latest/tools/spatial-analyst-toolbox/focal-statistics.htm and http://desktop.arcgis.com/en/arcmap/latest/tools/spatial-analyst-toolbox/raster-calculator.htm).

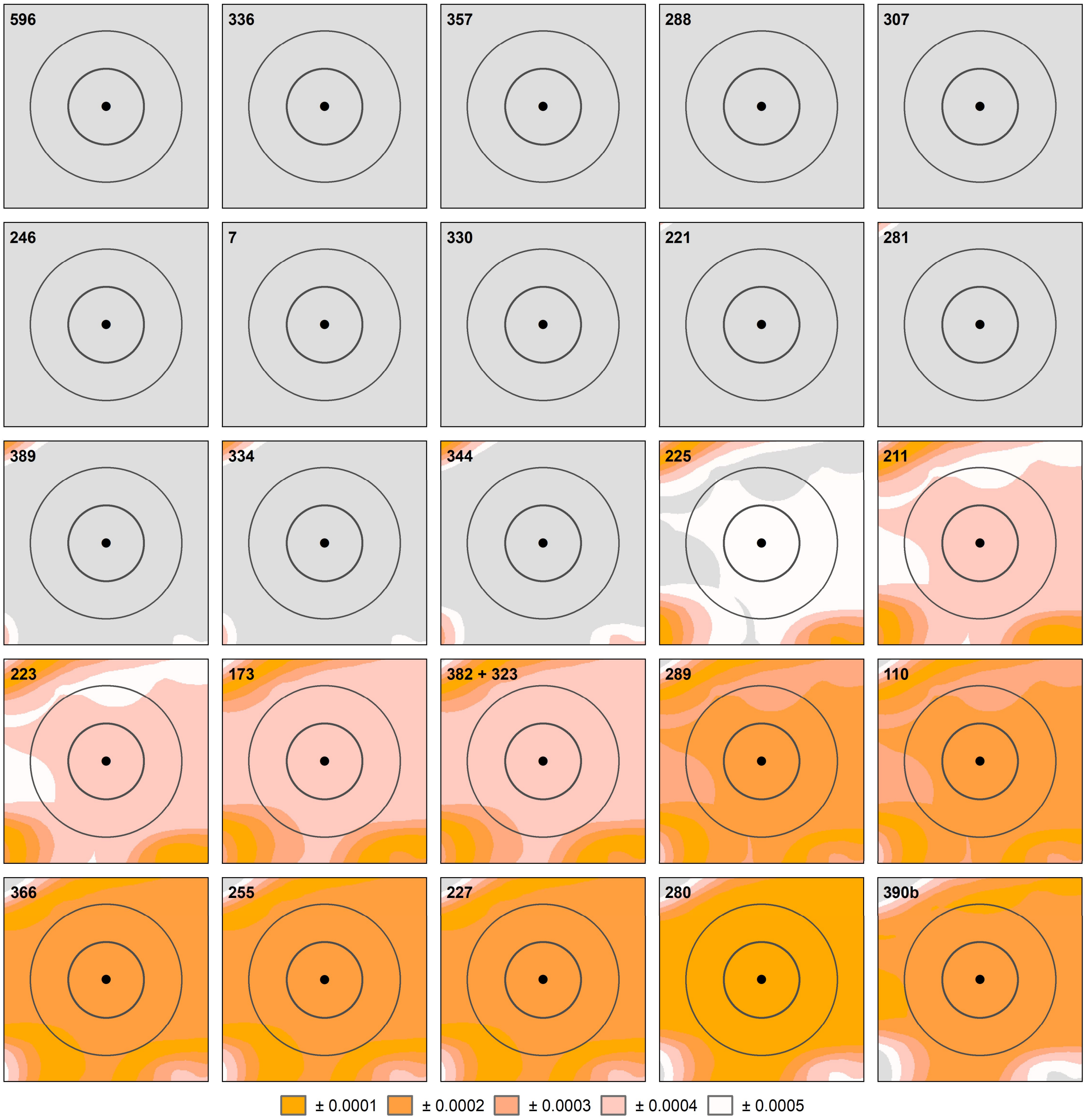

Supplement: Supplementary file 1 — Supplementary Material [file 41598_2018_28969_MOESM1_ESM.pdf]
